# Using targeted genome methylation for crop improvement

**DOI:** 10.1093/jxb/eraf131

**Published:** 2025-03-20

**Authors:** Zhibo Wang, Rebecca S Bart

**Affiliations:** Donald Danforth Plant Science Center, St. Louis, MO 63132, USA; Donald Danforth Plant Science Center, St. Louis, MO 63132, USA; Cardiff University, UK

**Keywords:** Epigenetics, plant stress, targeted epigenome modification

## Abstract

Genome editing allows scientists to specifically change the DNA sequence of an organism. This powerful technology now fuels basic biology discovery and tangible crop improvement efforts. There is a less well understood layer of information encoded in genomes, known collectively as ‘epigenetics’, that impacts gene expression, without changing the DNA sequence. Epigenetic processes allow organisms to rapidly respond to environmental fluctuation. Like genome editing, recent advances have demonstrated that it is possible to edit the epigenome of a plant and cause heritable phenotypic changes. In this review, we aim to specifically consider the unique advantages that targeted epigenome editing might provide over existing biotechnology tools. This review is aimed at a broad audience. We begin with a high-level overview of the tools currently available for crop improvement. Next, we present a more detailed overview of the key discoveries that have been made in recent years, primarily using the model system Arabidopsis, new efforts to extend targeted methylation to crop plants, the current status of the technology, and the challenges that remain to realize the full potential of targeted epigenome editing. We end with a forward-looking commentary on how epi-alleles might interface with breeding programs across a variety of crops.

## Teaser

We review the progress to date in developing and using targeted epigenome modification technologies.

## Introduction

Developing crops to withstand the impacts of climate change may require a change of thinking. Global climate change, along with the rapidly growing human population and increasing demand for nutritious food, has created an urgent need for significant advancements in crop improvement. Between 1880 and 2012, land and ocean temperature data show an average global warming of 0.85 °C, with projections suggesting an additional increase of 1.5 °C by 2050 (Intergovernmental Panel on Climate Change, 2014). Atmospheric concentrations of greenhouse gases, including carbon dioxide (CO_2_), methane (CH_4_), and nitrous oxide (N_2_O), have also significantly increased, with recent net emissions nearing 300 ppm (Intergovernmental Panel on Climate Change, 2014). These climatic changes are impacting the Earth’s surface, causing irregular and unpredictable precipitation patterns, higher temperatures, and the expansion of areas experiencing flooding or drought. At the same time, the global population is projected to increase by nearly 2 billion people over the next 30 years, rising from the current 8 billion to 9.7 billion by 2050, and potentially peaking at nearly 10.4 billion in the mid-2080s (United Nations, 2025). Ensuring that future generations have access to nutritious food is critical.

Traditionally, plant scientists have focused on developing crop varieties that are locally adapted to the specific stresses associated with different agroecological conditions. Crop varieties adapted to the Southwest USA probably show excellent drought tolerance while those developed for planting in the Southeast USA are likely to prioritize flooding tolerance, especially early in the season (Cart, 2021; McGeeney, 2021; Veit, 2023; Owens, 2024). However, current predictions for the effects of climate change suggest that our crops will increasingly face highly variable stresses (Rosenblatt and Schmitz, 2014; van der Wiel and Bintanja, 2021). A crop that experiences flooding in one year, may experience drought the following. Or worse, a single cropping season may experience unpredictable flooding early in the season and drought later in the season and then the reverse pattern the next year. Constitutive expression of any given stress response has a fitness cost (Geisel, 2011), and so the idealized crop variety of the future must be able to sense and respond to acute stresses as they occur. As such, plant scientists are likely to need to change their focus from developing highly productive crop varieties adapted to specific agroecological conditions to focus on developing crops that can flexibly respond to a myriad of different biotic and abiotic stresses, as they occur. Given their fluidity, epigenetic processes may be key to achieving this goal.

## Biotechnology tools for crop improvement

Over the past few decades, significant advancements in biotechnology, coupled with genomic sequencing, have transformed crop improvement by enabling precise genome modifications. The sequencing of the Arabidopsis genome in 2000 marked the beginning of this revolution (Arabidopsis Genome Initiative, 2000) and, to date, >4600 plant genomes have been sequenced, providing invaluable resources for functional genomics (Bernal-Gallardo and de Folter, 2024). Early genetic modification methods relied on T-DNA insertion via *Agrobacterium tumefaciens* or particle bombardment, allowing stable gene expression but with random genomic integration. While this approach has been instrumental in generating mutant collections, it is time-intensive and impractical for many economically significant crops. As alternatives, virus-induced gene silencing (VIGS) and RNAi have been widely used for gene function studies, with VIGS offering transient gene silencing and RNAi providing heritable modifications in transformed plants (Baulcombe, 1999; Agrawal *et al.*, 2003).

The development of targeted genome editing technologies brought greater precision to genetic modifications. Early approaches such as Zinc Finger Nucleases (ZFNs) and Transcription Activator-Like Effector Nucleases (TALENs) allowed targeted mutagenesis, but were limited by off-target effects and complex design requirements (Christian *et al.*, 2010; Osakabe and Osakabe, 2015). The introduction of CRISPR/Cas9 [clustered regularly interspaced palindromic repeats (CRISPR)/CRISPR-associated protein 9] in 2012 revolutionized genome editing by providing a simple, highly efficient system for precise gene modifications (Jinek *et al.*, 2012). CRISPR technology has since been widely applied across numerous crop species, including soybean (Wang *et al.*, 2023), rice (Zeng *et al.*, 2019; Zheng *et al.*, 2021), wheat (Sánchez-León *et al.*, 2024), maize (Tian *et al.*, 2024), tomato (Li *et al.*, 2022), citrus (Huang *et al.*, 2023), cassava (Elliott *et al.*, 2024; Lin *et al.*, 2025, Preprint), and banana (Shao *et al.*, 2020) to improve traits such as yield, stress tolerance, disease resistance, and nutritional content.

## What is DNA methylation in epigenetics

The term epigenetics is often attributed to C.H. Waddington who in 1942 published his thoughts on the topic as follows: ‘We certainly need to remember that between genotype and phenotype, and connecting them to each other, there lies a whole complex of developmental processes. It is convenient to have a name for this complex: “epigenotype” seems suitable’. (Waddington, 2012). Various epigenetic processes have now been defined and generally encompass heritable changes in gene expression that do not involve alterations to the underlying DNA sequence (Bird, 2007). These changes are often mediated by molecular mechanisms such as DNA methylation, histone modifications, and RNA-associated silencing, which can regulate gene expression without changing the genetic code itself. Briefly, histone modifications, such as methylation and acetylation, alter the structural configuration of chromatin and thus play a key role in controlling gene accessibility and expression. Additionally, non-coding RNAs can guide protein complexes to specific genome regions to modulate gene expression, adding another layer of epigenetic regulation. In this review, we focus on DNA methylation, one of the most well-understood epigenetic mechanisms, and explore its potential and applications in crop improvement. DNA methylation in plants is a dynamic and reversible process, with the observed methylation patterns being the result of a balance between methylation, maintenance, and demethylation activities.

### *De novo* establishment

DNA cytosine methylation in plants occurs in the CG, CHG, and CHH sequence contexts (where H represents any nucleotide except G) and is a key mechanism for gene regulation, either repressing or activating expression of nearby genes or transposable elements. *De novo* DNA methylation can be established through two pathways: the canonical RNA-directed DNA methylation (RdDM) pathway and the non-canonical RdDM pathway. The main difference between these pathways is how the siRNAs are produced. In the canonical RdDM pathway, siRNAs are generated by the RNA polymerase IV (Pol IV)–RNA DEPENDENT RNA POLYMERASE 2 (RDR2) complex and processed by the RNase III endonuclease DICER-LIKE 3 (DCL3). In the non-canonical RdDM pathway, a small amount of siRNAs can be produced from other sources, including inverted repeats, miRNA precursors, fragments of cleaved mRNA, or other non-coding RNAs that are not generated by Pol IV, through the coordination of RDR6 and DCL2 (Nuthikattu *et al.*, 2013; Cuerda-Gil and Slotkin, 2016). These siRNAs are loaded into ARGONAUTE 4 (AGO4) and AGO6, which guide the DOMAINS REARRANGED METHYLTRANSFERASE (DRM2), recruited by Pol V, to specific DNA sites and are crucial for initiating *de novo* RdDM at new locations (Erdmann and Picard, 2020; Sigman *et al.*, 2021).

### Methylation maintenance

The rules governing the maintenance of DNA methylation, especially across generations, determine its potential in practical applications. DNA methylation is a heritable mark passed down through both mitosis and meiosis (Law and Jacobsen, 2010). At CG sites, DNA methylation is maintained by DNA METHYLTRANSFERASE 1 (MET1), which interacts with VARIANT IN METHYLATION proteins (VIM1, VIM2, and VIM3) that bind to methylated cytosines on the parent strands (Woo *et al.*, 2007, 2008). Non-CG methylation, particularly in heterochromatic regions, is primarily maintained by plant-specific CHROMOMETHYLASE 2 (CMT2) and CMT3 (Zhang *et al.*, 2018). This maintenance occurs through a feedback loop with H3K9me2 histone modification and via the RdDM pathway in euchromatic regions (Du *et al.*, 2012; Stroud *et al.*, 2013, 2014).

### Demethylation

DNA demethylation contributes to the regulation of gene expression, and it can occur either passively or actively. Passive demethylation primarily takes place during DNA replication when the newly synthesized DNA strand is not methylated by DNA methyltransferases. Active demethylation involves the direct removal of methyl groups by DNA demethylases. In Arabidopsis, four DNA demethylases have been identified: DEMETER (DME), DME-Like 1/Repressor of Silencing 1 (DML1/ROS1), DML2, and DML3 (Morales-Ruiz *et al.*, 2006; Penterman *et al.*, 2007; Ortega-Galisteo *et al.*, 2008; Kumar *et al.*, 2018).

## DNA methylation associated with crop performance

While sequencing plant genomes has become common place, elucidating the full ‘methylome’ is still a time-consuming and costly endeavor. However, for species where the epigenome has been characterized, these data have deepened our understanding of plant functional genomics, genome evolution, and domestication processes, and also revealed genetic factors that influence responses to environmental changes and pathogen resistance (Peng *et al.*, 2023). Epigenetic marks in plants, including DNA methylation, can be influenced by environmental factors and cause changes in gene expression. Thus, these mechanisms play a vital role in crop adaptation. Here, we will focus on one specific epigenetic mark, methylation. Currently, two primary strategies are used to elucidate the effects of DNA methylation on gene expression and phenotypes. These have been reviewed extensively elsewhere (He *et al.*, 2011; Pikaard and Mittelsten Scheid, 2014). In brief, the first approach involves integrating methylome sequencing with transcriptome data to identify differentially methylated regions (DMRs) associated with gene expression variation. This method can establish links between DNA methylation, gene expression, and physiological outcomes, especially when coupled with observed changes in physiological characteristics under various experimental treatments, such as biotic or abiotic stress. The second approach involves knocking out one or more of the known DNA methyltransferases, resulting in a reduction in DNA methylation across the genome. Researchers may then identify epimutations and associate them with specific traits. In the sections below, we summarize some of the variation in DNA methylation that has been linked to abiotic and biotic stress resistance, and to plant development. Our literature search focused primarily on discoveries reported in the last 5 years.

### Abiotic stress

Recent publications have established a connection between epigenetic marks and abiotic stresses, including drought, temperature, and salt stresses. In barley, the DNA demethylase gene DEMETER (HvDME) responds to water deficiency. Analysis of the HvDME promoter region revealed two distinct areas with different methylation patterns: a proximal region near the translation start site with low methylation and a distal region with high methylation enriched in both CG and non-CG methylation. Under drought stress, methylation changes occurred in the distal region, while the proximal region remained unaffected (Drosou *et al.*, 2021). Plant hormone regulation has also been implicated in modulating DNA methylation during drought stress. In an abscisic acid (ABA)-deficient maize mutant, drought stress causes significant changes in DNA methylation, notably a decrease in CG and CHG methylation levels. This altered methylation pattern may affect stress-responsive gene expression, providing a potential mechanism for drought adaptation despite insufficient ABA production, which is crucial for drought tolerance (Sallam and Moussa, 2021).

In addition to water scarcity, researchers have studied how DNA methylation responds to temperature variations. In sweet cherry, certain genes influenced by DNA methylation are implicated in chilling accumulation (Rothkegel *et al.*, 2020), while in cotton, genes responsive to heat stress are believed to support normal anther development under high temperature conditions (Zhang *et al.*, 2020). A study on *Brassica napus* revealed different DNA methylation patterns in heat-sensitive and heat-tolerant genotypes under heat stress, with the heat-tolerant genotype exhibiting predominantly hypomethylated DNA, suggesting that methylation plays a role in *B. napus* heat stress response and adaptation (Gao *et al.*, 2014). A nucleoproteome analysis of *Pinus radiata* under high-temperature stress revealed a decrease in the abundance of *S*-adenosylmethionine (SAM) synthase and *S*-adenosyl-l-homocysteine hydrolase (SAHH), leading to altered DNA methylation profiles. SAM serves as a crucial cofactor and methyl group donor, while SAHH is essential for regenerating the SAM during methylation-mediated gene silencing, providing evidence of the key role of DNA methylation in heat stress tolerance and priming mechanisms (Lamelas *et al.*, 2020).

DNA methylation plays a vital role in regulating plant responses to salt stress and nutrient deficiencies. The high-affinity potassium (K^+^) transporter (HKT1) gene is a crucial salt tolerance gene in rice that encodes an Na^+^ transporter, which helps remove Na^+^ from leaves and maintain Na^+^/K^+^ balance. Under salt stress, increased methylation in the CHH and CHG contexts within miniature inverted-repeat transposable elements (MITEs) was observed for this gene, leading to its activation and subsequently enhancing salt tolerance (Wang *et al.*, 2020). Meyer *et al.* (2019) demonstrated that RDR2 plays a role in biomass accumulation under nitrogen deficiency in *Arabidopsis thaliana*, suggesting that the RdDM pathway may be involved in regulating N deficiency responses. Yong-Villalobos *et al.* (2015) reported that phosphorus (P) starvation induces genome-wide DNA methylation changes in *A. thaliana*, accompanied by alterations in gene expression. Their study revealed that P deficiency led to the up-regulation of DMRs in 20% of shoot-associated genes and 86% of root-associated genes. They concluded that DNA methylation modifications are essential for regulating P-sensitive genes and are necessary for establishing physiological and morphological responses to P starvation

### Biotic stress

Plants have evolved sophisticated defense mechanisms to withstand pathogens and pests, which has been extensively studied and reviewed over the past two decades (Jones *et al.*, 2016; Zhou and Zhang, 2020). A growing body of research has revealed that epigenetic regulation plays a crucial role in shaping plant immunity and driving phenotypic variation during plant–microbe interactions. However, the role of DNA methylation in plant immunity is complex, with effects that can lead to either resistance or susceptibility, depending on the type of pathogen.

Since Arabidopsis mutants with DNA hypomethylation are relatively easy to access, their responses to biotic stress have been extensively studied to provide insights into the role of DNA methylation in plant immune responses. Mutants such as *met1*, *drm2*, *cmt3*, *nrpd1* (Pol IV mutant), *nrpe1* (Pol V mutant), *rdr2*, and *dcl2/3*, which exhibit hypomethylation, show greater resistance to both the bacterial pathogen *Pseudomonas syringae* pv. *tomato* (*Pst*) and the obligate biotrophic oomycete pathogen *Hyaloperonospora arabidopsidis* (*Hpa*), where these genes function either coordinately or independently (López Sánchez *et al.*, 2016; Cambiagno *et al.*, 2021). Conversely, some mutations affecting *de novo* methylation and its maintenance increase susceptibility to pathogen infection. For example, the *rdr6* and *dcl2/3/4* mutants, which significantly reduce the biogenesis of siRNAs, are more susceptible to the fungal pathogen *Botrytis cinerea* (Cai *et al.*, 2018). Mutants of *nrpe1* and *nrpd2* also show enhanced susceptibility to the necrotrophic fungus *Plectosphaerella cucumerina* (López Sánchez *et al.*, 2016), and the triple mutant *drm1/drm2/cmt3* (*ddc*) is more susceptible to the necrotrophic fungus *Alternaria brassicicola* (López *et al.*, 2011; Luna *et al.*, 2012). This increased susceptibility is due to the suppression of jasmonic acid (JA)-dependent defense signaling against *P. cucumerina* and *A. brassicicola* in the *nrpe1* and *ddc* mutants. DNA methylation within repetitive sequences or transposable elements (TEs) has been reported to be altered in response to infection by nematode, bacterial pathogen *Pst*, and salicylic acid (SA) treatment, subsequently regulating the transcription of nearby genes (Agorio and Vera, 2007; Dowen *et al.*, 2012; Rambani *et al.*, 2015).

Interestingly, components of both the canonical and non-canonical RdDM pathways are involved in plant responses to biotic stress, highlighting the diverse role of epigenetic regulation in coping with biotic challenges. Additionally, active demethylation also plays a role in transcriptional reprogramming of immune response genes upon pathogen infection. For instance, ROS1 antagonizes the RdDM pathway, which is mediated by the bacterial flagellin peptide flg22, at the locus of *Resistance methylated gene 1* (*RMG1*), a basal *Pst* resistance gene, contributing to antibacterial defense (Halter *et al.*, 2021). The *ros1* mutant shows hypermethylation and is more susceptible to *Hpa*. Conversely, *ros1* displays increased resistance to *P. cucumerina*, which is linked to JA-dependent defense pathways (López Sánchez *et al.*, 2016). Furthermore, the tissue-specific expression of four DNA demethylases—DME, ROS1, DML2, and DML3—acts cooperatively to build resistance against the hemibiotrophic vascular fungal pathogen *Fusarium oxysporum* (Schumann *et al.*, 2019).

Epigenetic changes triggered by biotic stress can sometimes be passed down to subsequent generations, resulting in transgenerational priming (López Sánchez *et al.*, 2021; Morán-Diez *et al.*, 2021). In Arabidopsis, transgenerational acquired resistance was observed after three generations of exposure to *Pst*, with notable changes in DNA methylation at CG sites within gene bodies (Stassen *et al.*, 2018). Additionally, a population of epigenetic recombinant inbred lines (epiRILs) was screened for resistance to Hpa. These lines, while sharing the same genetic background, exhibit variations in heritable DNA methylation patterns. Phenotypic analysis and RNA-sequencing showed that *Hpa*-resistant epiRILs are primed to initiate robust, inheritable defense responses at the early stages of infection (Furci *et al.*, 2019). Therefore, DNA methylation changes in previous generations of Arabidopsis may contribute to transgenerational acquired resistance.

### Plant development

The relationship between DNA methylation and plant development has also been explored in various studies, and this topic has been reviewed extensively (Gady *et al.*, 2017; Brukhin and Albertini, 2021; Wang and Yamaguchi, 2024). DNA methylation plays a pivotal role in various aspects of flower development, including anthocyanidin deposition and floral patterning. For example, the *SUPERMAN* (*sup*) gene is involved in flower development in Arabidopsis and is subject to epigenetic regulation. Several *sup* epialleles (known as the *Clark Kent* alleles) have been described with various levels of generational stability (Jacobsen and Meyerowitz, 1997). In the orchid *Oncidium*, DNA methylation was found to regulate anthocyanin accumulation. In the ‘White Jade’ (WJ) variety of *Oncidium*, the absence of methylation in the promoter of *OgCCD1* (*Carotenoid Cleavage Dioxygenase 1*), which catabolizes carotenoid metabolites, leads to its active expression, resulting in carotenoid degradation and the development of white flowers (Chiou *et al.*, 2010). Furthermore, genome-wide sulfite sequencing revealed that most differentially methylated genes were involved in several steps of the benzoylalanine biosynthesis pathway, responsible for producing >90% of floral volatiles during *Prunus mume* floral development (Yuan *et al.*, 2021). The occurrence of non-infectious bud failure (NBF), a physiological disorder common in almonds but absent in peaches, has also been linked to DNA methylation (D’Amico-Willman *et al.*, 2022). Additionally, DNA methylation levels fluctuate dynamically during seed development and germination. Specifically, CHH methylation increases during Arabidopsis seed development but decreases during germination, primarily due to passive demethylation, indicating its involvement in regulating seed dormancy (Walker *et al.*, 2018). Interestingly, while maize male reproductive lineages exhibit lower overall CHH methylation than somatic cells, certain hypermethylated CHH sites are essential for meiosis (Zhang *et al.*, 2014).

DNA methylation is a key regulator influencing vegetative development and phenotypic characteristics. Dysfunction of ROS1 (REPRESSOR OF SILENCING 1), a DNA glycosylase/lyase that demethylates DNA and regulates gene expression, leads to hypermethylation of the *AtEPF2* promoter, suppressing the expression of stomatal inhibitory factors and causing excessive stomatal production in Arabidopsis (Yamamuro *et al.*, 2014). In grapevine, the combination of RNA-seq and whole-genome bisulfite sequencing identified a hypo-DMR within *ACO1*, an *ACC* (*aminocyclopropanecarboxylate*) *oxidase* gene involved in the ethylene signaling pathway that may contribute to self-pruning, an important horticultural trait (Zou *et al.*, 2020). Hypomethylation was indicated in potato to promote tuber initiation in strict short-day genotypes by regulating key genes involved in photoperiod and gibberellic acid responses (Ai *et al.*, 2021). To comprehensively investigate the role of DNA methylation in plant development, He *et al.* (2022) knocked out all five known DNA methyltransferases in Arabidopsis, resulting in plants entirely lacking DNA methylation. This quintuple mutant exhibited a range of developmental defects, highlighting the importance of DNA methylation for various aspects of plant development.

### Artificially induced epialleles

In addition to naturally occurring epialleles, an alternative approach to investigating the impact of DNA methylation on desirable crop traits involves artificially inducing epigenetic variation. In Arabidopsis, epimutants are created in genetic backgrounds where DNA methylation is disrupted. The genes *MET1* and *DECREASE IN DNA METHYLATION 1* (*DDM1*) encode a maintenance DNA methyltransferase and a SWI/SNF-like ATP-dependent chromatin remodeling factor, respectively (Vongs *et al.*, 1993; Jeddeloh *et al.*, 1998). Mutants for *met1* and *ddm1* show reduced DNA methylation across the genome, leading to heritable epimutations that cause phenotypic changes. For example, in a *ddm1* mutant background, the *FLOWERING WAGENINGEN* (*FWA*) epimutant experiences a loss of DNA methylation upstream of the coding sequence in repeat regions, resulting in the up-regulation of *FWA* transcription and delayed flowering (Soppe *et al.*, 2000). By mutating *DDM1*, a syndrome called bonsai is caused by hypermethylation and silencing of the *Anaphase-Promoting Complex 13* (*APC13*) gene. This gene silencing is mediated by a nearby long interspersed nuclear element (LINE) retrotransposon, demonstrating a paradoxical hypermethylation in the *ddm1* mutant that typically induces hypomethylation elsewhere in the genome (Saze and Kakutani, 2007).

This ‘sledgehammer approach’ strategy has been applied to other plant species as well. In tomatoes, the *ddm1b* mutant displayed reduced sensitivity to heat stress, which was associated with changes in gene expression (Singh *et al.*, 2021). In addition to using *ddm1* or *met1* mutants as a tool, fine-tuning the expression of genes involved in *de novo* establishment and maintenance of methylation, as well as demethylation, through knockdown or overexpression, has also demonstrated significant roles in plant growth, development, and response to environmental stimuli. For instance, RNAi-mediated silencing of the *DDM1* gene in poplar enhanced resistance to drought-induced cavitation (Sow *et al.*, 2021). Silencing of pepper *MET1-like1* by VIGS led to DNA hypomethylation, increased content of soluble solids, and accumulation of carotenoids in the fruit, which was accompanied by changes in expression of genes involved in capsanthin/capsorubin biosynthesis, cell wall degradation, and phytohormone metabolism and signaling (Xiao *et al.*, 2020). In rice, overexpression of OsAGO2, a key component in *de novo* methylation, increased rice susceptibility to rice black-streaked dwarf virus (RBSDV) infection, while *ago2* mutant lines showed strong resistance by triggering an early defense response, including the up-regulation of defense genes and production of reactive oxygen species (ROS). In the *ago2* mutants, RBSDV infection led to decreased methylation at the *HEXOKINASE 1* (*OsHXK1*) promoter and its significant increased expression. Overexpression of OsHXK1 in rice also induced ROS production and enhanced resistance to RBSDV (Wang *et al.*, 2021). These findings suggest that *OsHXK1* regulates ROS accumulation and is controlled by *OsAGO2* through epigenetic mechanisms. RNA methylation in apple (*Malus domestica*) has been manipulated by either overexpressing or suppressing *MdMTA*, a catalytically active component of the m^6^A methyltransferase complex. This approach has further confirmed the role of MdMTA as a positive regulator of drought tolerance in apple trees by modulating the mRNA stability and translation efficiency of genes involved in lignin metabolism and oxidative stress response (Hou *et al.*, 2022).

## Targeted DNA methylation editing of the Arabidopsis *fwa* gene and advances in editing methods

**Fig. 1.**
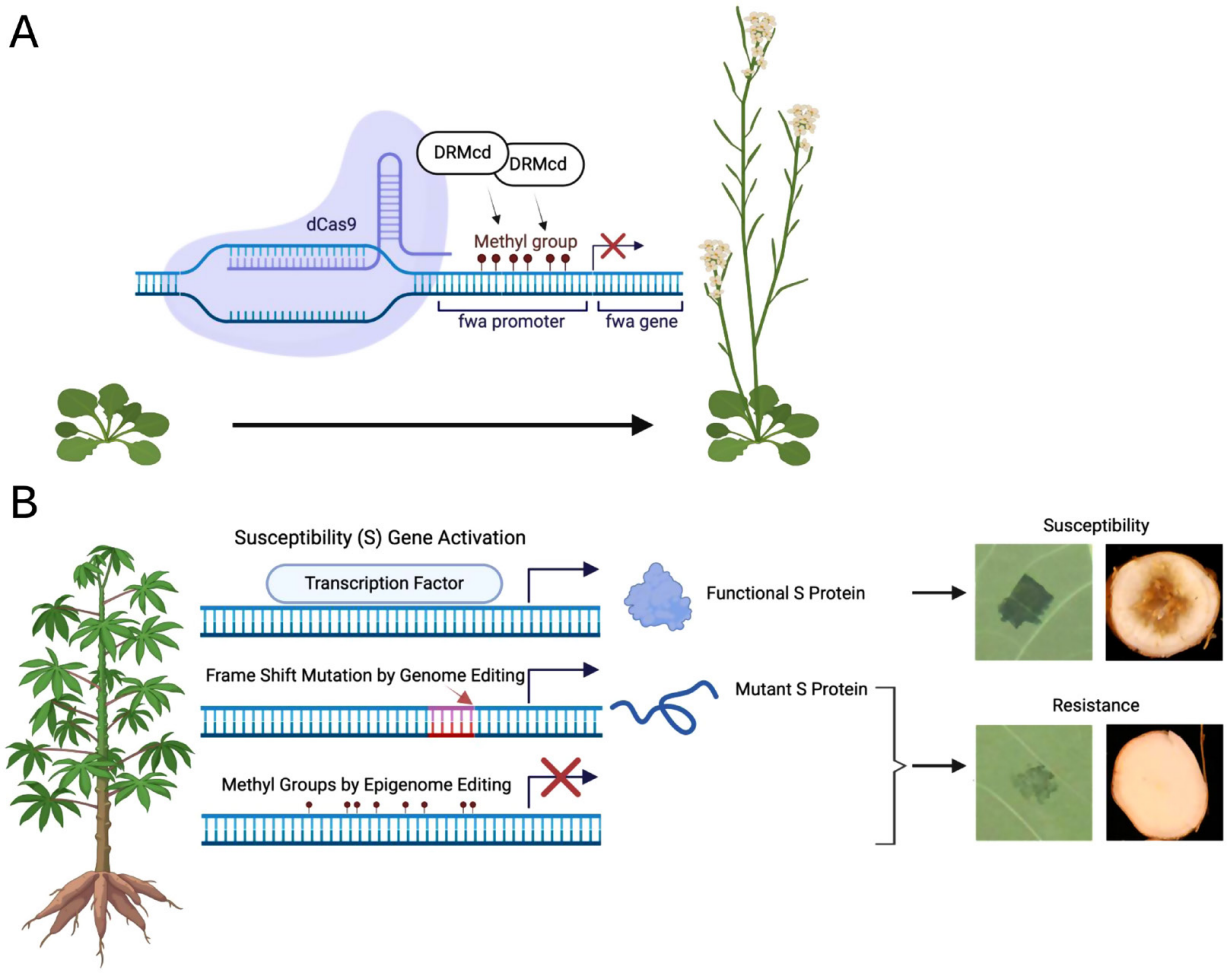
Targeted DNA methylation in plants can alter phenotypes and offers potential for crop improvement. (A) Methylation of the *FWA* gene suppresses its expression, thereby promoting flowering in Arabidopsis (Soppe *et al.*, 2000; Ghoshal *et al.*, 2021). To achieve this, the CRISPR/Cas9 system has been modified by deactivating Cas9 into dCas9, which retains its DNA binding capability but lacks nuclease activity. When paired with DRMcd, a methyltransferase, this system enables precise, targeted DNA methylation in plants. (B) Methylation of a susceptibility (S) gene can suppress its expression, thereby enhancing disease resistance in cassava. Targeted DNA methylation blocks the promoter region of the S gene, preventing its activation. Unlike gene editing, targeted methylation does not introduce mutations into the DNA sequence. In this context, the disease phenotype refers to cassava bacterial blight, caused by *Xanthomonas phaseoli* pv. *manihotis* (Veley *et al.*, 2023) and could potentially also be effective at suppressing necrosis of the storage roots caused by cassava brown streak virus (Gomez *et al.*, 2019; Lin *et al.*, 2025). Created in BioRender. Bart, R. (2025) https://BioRender.com/d27j441.

The previous sections illustrate the various phenotypic consequences of epigenetic regulation, both naturally occurring and artificially induced. Over the last decade, scientists have worked to develop new tools to intentionally modify methylation patterns at specific places in the genome. A pioneering attempt in Arabidopsis involved the integration of a zinc finger (ZF) protein with the RdDM pathway enzyme, SUVH9 (Johnson *et al.*, 2014). This integration was effective in promoting DNA methylation at the previously unmethylated epiallele of *fwa-4* through the RdDM pathway, resulting in the silencing of the *FWA* gene and an early flowering phenotype. The study further confirmed that these methylation modifications were reliably inherited, even in the absence of any transgene component. A subsequent study leveraged a ZF-targeting strategy to investigate how other RdDM elements contribute to *de novo* DNA methylation (Gallego-Bartolomé *et al.*, 2019). This study revealed that dual targeting of RNA Pol IV and RNA Pol V complexes could substantially enhance the targeted methylation process. Beyond the standard plant RdDM machinery, a fusion of *Spiroplasma* sp. strain MQ1 CG methyltransferase SssI with the ZF protein was shown to establish inheritable CG methylation at the FWA promoter, effectively repressing its expression (Liu *et al.*, 2021). Yet, systems expressing these ZF fusions with both SssI and RdDM components displayed genome-wide unspecific hypermethylation. To minimize off-target hypermethylation, a modified CRISPR/dCas9 system used a variant of MQ1 (MQ1v) with decreased DNA affinity (Ghoshal *et al.*, 2021). This modification, compared with the combination of ZF with SssI, led to inheritable DNA methylation at the FWA promoter with reduced off-target impacts. Additionally, employing the SunTag system in conjunction with CRISPR/dCas9 boosted the methylation accuracy at the *FWA* target (Ghoshal *et al.*, 2021) (Fig. 1A). Moreover, the SunTag–CRISPR/dCas9 system, when merged with the catalytic domain of the *Nicotiana tabacum Domains Rearranged Methyltrasferase 2* (*DRM2*) catalytic domain (NtDRMcd), targeted methylation precisely to the *FWA* promoter (Papikian *et al.*, 2019). These investigations underscore that various DNA methyltransferases, when paired with DNA-binding modules, are capable of inducing inheritable methylation at specific targets. However, occasional instances of partial inheritance and off-target effects underscore the necessity for an effective and accurate DNA-binding module to ensure successful DNA methylation manipulation.

On the other hand, in plants, examples of targeted DNA demethylation efforts are sparse. To date, only one instance has been documented where the human ten-eleven translocation dioxygenase (TET1) catalytic domain (TET1cd) was utilized, either paired with a ZF protein or integrated into the SunTag–CRISPR/dCas9 system (Gallego-Bartolomé *et al.*, 2018). When linked to a ZF protein aimed at the *FWA* promoter—termed ZFFWA–TET1cd—this fusion protein reduced DNA methylation at the promoter, thereby inducing a late-flowering phenotype. Remarkably, this demethylation effect persisted even when the transgene module for DNA demethylation was segregated out (Gallego-Bartolomé *et al.*, 2018). Furthermore, targeting the CACTA1 transposon within heterochromatic regions using ZF–TET1cd (hereafter called ZFCACTA1–TET1cd) not only decreased DNA methylation but also reactivated transcription elements. In contrast, without the presence of ZFCACTA1–TET1cd, methylation at CACTA1 was reinstated, and its silencing resumed (Gallego-Bartolomé *et al.*, 2018). This suggests that removing DNA methylation incompletely in heterochromatic areas may re-attract the RdDM apparatus, resulting in the restoration of methylation. Moreover, the SunTag–CRISPR/dCas9–TET1cd approach was reported to prompt DNA demethylation at both *FWA* and *CACTA1* sites with minimal side effects. Compared with ZFCACTA1–TET1cd, which caused widespread hypomethylation, the SunTag–CRISPR/dCas9–TET1cd achieved more precise demethylation at target sites (Gallego-Bartolomé *et al.*, 2018). Notably, distinct enzymes are involved in DNA demethylation in mammals and plants. In mammals, TET family proteins catalyze the conversion of 5-methylcytosine (5mC) into 5-hydroxymethylcytosine (5hmC) (Tahiliani *et al.*, 2009; Ito *et al.*, 2010). Conversely, plants employ the DEMETER (DME) family of proteins, which directly eliminate 5mC (Gehring *et al.*, 2006). This suggests that incorporating DME into the SunTag system might be a better fit for targeted DNA demethylation editing in plants.

## Epigenome editing for crop improvement

Beyond Arabidopsis, epigenome editing has recently been described for the important food security crop, cassava (*Manihot esculenta*) (Veley *et al.*, 2023) (Fig. 1B). Cassava is susceptible to *Xanthomonas phaseoli* pv. *manihotis* (*Xpm*), the causal agent of bacterial blight. In this pathosystem, *Xpm* delivers the transcription activator-like (TAL) protein, TAL20, to plant cells where it activates transcription of the *MeSWEET10a* susceptibility gene through direct promoter binding. Building from the epigenome editing tools developed in Arabidopsis, a synthetic ZF DNA-binding domain was fused to a component of the RdDM pathway, DMS3. This fusion was directed to the promoter of *MeSWEET10a* to induce DNA methylation which successfully inhibited the binding of the TAL20 protein and prevented the transcriptional activation of *MeSWEET10a* during infection. Consequently, this reduced the expression of the susceptibility gene and led to diminished symptoms of bacterial blight in cassava plants (Fig. 1B). Little to no off-target methylation was observed, and the methylation did not negatively impact cassava’s growth and development, indicating that this epigenome editing approach could enhance disease resistance without compromising plant health.

Plant pathogenic bacteria from the *Xanthomonas* and *Ralstonia* genera are globally distributed and cause diseases in many economically significant mono- and dicot plants including pepper, tomato, citrus, rice, soybean, sugarcane, banana, and eggplant (Peeters *et al.*, 2013; An *et al.*, 2020). Like *Xpm*, many of these pathogens use TAL effectors to induce expression of cognate susceptibility genes in their host plants (Antony *et al.*, 2010; Cohn *et al.*, 2014; Hu *et al.*, 2014; Cox *et al.*, 2017; Phillips *et al.*, 2017). Based on the results in cassava, using targeted DNA methylation to block access to susceptibility genes may be a broadly applicable crop protection strategy.

## Conclusion

Throughout this review, we have looked at existing technologies used for crop improvement and introduced targeted epigenome editing as a potential new strategy. In this final section, we attempt to answer two important questions. First, with so many existing crop improvement strategies online, what are the potential benefits of targeted genome methylation? And second, how would newly developed ‘epi-traits’ be integrated into breeding pipelines?

### What are the potential benefits of targeted genome methylation?

Gene editing, an incredibly powerful technology, has primarily been used to introduce knockout mutations into genes of interest. However, this strategy may not work in situations where complete knockout of a gene results in lethality or severe developmental defects. More subtle gene expression changes may be achieved through edits to regulatory elements, but these are difficult to predict. Similarly, RNAi and VIGS can achieve variable gene expression patterns, but this is a relatively uncontrolled, stochastic process, depending on expression of the transgene in the case of RNAi, and the virulence of the virus in the case of VIGS. Furthermore, VIGS-induced gene expression is not heritable, and RNAi requires the presence of a transgene to suppress gene expression. It is also notable that epigenome editing does not alter the DNA sequence. While other genetic modification techniques introduce permanent changes to the genome, epigenome editing involves potentially reversible modifications that can regulate gene expression without changing the sequence. This characteristic may factor into the regulatory landscape surrounding future epi-edited crops.

In the face of these limitations with existing tools, epigenome editing may offer an easier option for fine-tuning gene expression through variable levels of promoter methylation. It has been reported that epigenome editing, particularly methylation, can be inherited by subsequent generations even in the absence of any transgene components (Johnson *et al.*, 2014; Gallego-Bartolomé *et al.*, 2018). Indeed, manipulating promoter methylation is a natural plant response to environmental changes, used to fine-tune gene expression like a dial rather than a simple switch.

### How would newly developed ‘epi-traits’ be integrated into breeding pipelines?

The rules governing the inheritance of epigenetic modifications are not yet fully understood. In plants, the only gene that has been artificially methylated and shown to be heritable is *FWA* in Arabidopsis. The recent work in cassava that demonstrated directed methylation to a susceptibility gene is promising, but heritability has yet to be demonstrated (Veley *et al.*, 2023). It is still too early to conclude that epigenetically edited alleles can be consistently inherited. Understanding the inheritance patterns and stability of epigenetic modifications is crucial for the effective application of epigenome editing in crop breeding, as it ensures the reliable transmission of these traits to subsequent generations.

Inheritance aside, if the justification of this work is for crop improvement, then we must also consider how the envisaged ‘epi-traits’ will be integrated into breeding pipelines. This may look different for different crops. For example, extreme stability through inheritance will probably be required for crops that are intensively bred, such as maize. Clonally propagated crops may find utility with less heritable epialleles so long as they are stable through mitotic cell division. Finally, it is worth noting that the true advantage of epialleles may be in their instability. With climate change, significant variation in weather patterns is expected. The value of epigenome editing may be in a future ability to create varieties that sense and respond to stresses only when needed. While the progress made over the last decade is impressive, as highlighted throughout this review, many gaps in our knowledge of epigenetics remain. Filling these gaps and reaping the benefits of novel strategies for crop improvement will require significant and sustained effort and funding.
